# Tissue Distribution of Mercury and Its Relationship with Selenium in Atlantic Bluefin Tuna (*Thunnus thynnus* L.)

**DOI:** 10.3390/ijerph182413376

**Published:** 2021-12-19

**Authors:** Antonio Belmonte, Pilar Muñoz, Juan Santos-Echeandía, Diego Romero

**Affiliations:** 1 TAXON Estudios Ambientales S.L. C/Uruguay s/n, 30820 Alcantarilla, Spain; antonio.belmonte@taxon.es; 2 Departamento de Sanidad Animal, Facultad de Veterinaria, Campus de Espinardo, Universidad de Murcia, 30100 Murcia, Spain; pilarmun@um.es; 3 Instituto Español de Oceanografía, Subida a Radio Faro, 50-52, 36390 Vigo, Spain; juan.santos@ieo.es; 4 Área de Toxicología, Facultad de Veterinaria, Campus de Espinardo, Universidad de Murcia, 30100 Murcia, Spain

**Keywords:** Atlantic bluefin tuna, farmed, Mediterranean Sea, mercury, selenium, tissues

## Abstract

Mercury (Hg) is an important heavy metal to consider in marine predators, while selenium (Se) has a natural antagonistic effect on this metal in fish. The Atlantic bluefin tuna (ABFT, *Thunnus thynnus*) is a pelagic top-level predator of the trophic web and their Hg muscular content is an object of concern in food safety. Nevertheless, little is known about levels of this metal in remaining tissues, which may be important as by-product source, and its relationship with Se. Thus, concentration of both elements in liver, kidney, brain, gill and bone, in addition to muscle, of ABFT were determined. The kidney was the tissue with the highest concentration of Hg (Total-Hg, THg) and Se, and the Se/THg concentration ratio was similar in all tissues, except bone and muscle. The Selenium Health Benefit Value (HBV_Se_) was positive in each specimen and tissue, indicating that the Se plays an important role against Hg not only in the muscle.

## 1. Introduction

The presence of heavy metals in marine ecosystems has increased in recent years, derived from natural and anthropogenic sources [1,2] continuously depositing in the seabed and interacting directly with the biota [3,4]. Mercury (Hg) is highly toxic, and its pollution is understood to be global, diffuse and a chronic problem [5]. This heavy metal is included in the Substance Priority List from the Agency for Toxic Substances and Disease Registry [6], as a priority substance in the field of water policy [7,8], as a priority pollutant under the clean water act [9] and subject to international agreements [10]. In addition, this non-essential metal produces important effects on animal and human health, so it has been a frequent object of research [11]. The global costs to society by neurocognitive deficits due to Hg toxicity are estimated at several USD billions [12,13].

Although Hg pollution is a worldwide problem in seas and oceans, it is especially important in the Mediterranean Sea by mining and sub-marine volcano activities [14], with at least three primary sources of atmospheric deposition in the surface sediments: urban, industrial and global precipitation-derived [15]. Over the last decades, fish from the Mediterranean Sea have been linked with higher Hg concentration than other oceans [16,17,18,19], becoming a problem of great concern as fish plays a fundamental role in the Mediterranean diet. This diet recommends consuming fish at least twice a week, in order to reduce risk of contracting many pathologies such as cardiovascular disease, lung disease, cancer or Alzheimer’s disease, thanks to the intake of omega-3 fatty acids content [20]. 

Accumulation and biomagnification of Hg in top-level aquatic predators is well known, being considered that environmental concentrations in predator’s tissues are amplified by a million times or more [21]. Atlantic bluefin tuna (ABFT, *Thunnus thynnus* L.) is a top pelagic predator of the marine food web. In the last years the potential risks associated with excessive consumption of tuna have been largely discussed [22,23,24]. The problem of bioaccumulation of methylmercury (MeHg) in marine predators is expected to be exacerbated by climate change [21], which could increase concerns about the potential health risks derived from the consumption of this species. Maximum levels for Hg in certain foods have been established by European authorities, 1.0 µg g^−1^ (wet weight) being the maximum level in muscle meat of Thunnus species [25]. In this regard, some recent reviews highlighted the need of more knowledge about this issue [26,27], with more attention to the toxicology in marine fish due to its bioaccumulation and toxic effects [28]. As a result, many studies have been focused on measuring the Hg content in muscle as well as in liver of marine predators that are frequently consumed by humans, recommended as an environmental indicator of water pollution, even though the muscle can show higher Hg concentration [29]. On the other hand, by-products of the tuna aquaculture industry (such as gill, guts and bones) go to animal feed (pet food, fish meal for aquaculture), industrial uses (essential oils and other derivatives), or for human consumption [30,31]. However, scientific literature reporting tissue distribution of Hg in other tissues of ABFT is very scarce [29,32,33,34].

Finally, Selenium (Se) is an antagonist of Hg toxicity, which protects tissues against oxidative damage and prevents pathological consequences of Hg bioaccumulation [35]. If the Se supply is altered, the fish does not respond adequately to growth and physiological and biochemical functions (such as antioxidant response), but an excess could also be harmful [36]. Teleost fishes transport through their body more Se than most other vertebrates [37], and have developed greater dependence on environmental Se. In addition, some predatory species contain higher concentrations of Se than those reported in other fish species [38]. However, Se is only studied in fish muscle for human food safety. In fact, it is reported that the Selenium Health Benefit Value (HBV_Se_ [39]) is a good instrument to better understand the available Se that remains after its interaction with Hg.

Thus, in light of the above, the aim of this study was to better understand the Hg and Se interaction in several tissues of ABFT from Mediterranean stock, since that could contribute to environmental knowledge in relation to this pollutant, its toxicokinetic properties, and the tuna health status by Hg exposure. Due to the lack of existing information regarding Hg tissular distribution, Total-Hg (THg) and Se concentration in gills, brain, kidney and bone in ABFT, in addition to liver and muscle, were assessed.

## 2. Material and Methods

### 2.1. Sample Collection

Samples (liver, kidney, muscle, brain, gill and bone) were taken from 43 specimens of ABFT that had been purse-seined in Balearic Sea (Mediterranean Sea) in May 2016 and transferred to sea cage culture facilities in San Pedro del Pinatar (Murcia, Spain) (37° 49′ 55.46″ N–0° 39′ 42.33″ W) where they were kept until slaughter for human consumption (September to October 2016). The specimens (length 202–248 cm, weight 206–360 kg, 27 males and 16 females) were shot underwater by lupara (shocking-death) then hoisted by crane, and the age was determined from the size [40]. The samples were carefully taken (0.5–1 g) according to the protocol established by the ICCAT through the Atlantic-Wide Research Programme for Bluefin Tuna. The muscle samples were dissected from the caudal peduncle, and bone samples were collected from the bony denticles of the branchial arch. Bone samples were dried at 40 °C to constant weight, and the dry matter percentages for each sample (specimen) were calculated. Gill samples were washed with purified water (MilliQ), nitric acid (2%), and again MilliQ water, and then dried at room temperature. All samples were stored at -20 °C until analysis.

### 2.2. Metal Analysis

The THg content in each tissue (samples in triplicate) was determined by using the pyrolysis atomic absorption spectrometry with gold amalgamation (AMA254 Advanced Mercury Analyzer, LECO Instrumentos S.A., Madrid, Spain), according to the methodology describe by Costley et al. [41]. In order to ensure quality of the results obtained, analytical blanks and certificate material (NIST2976) were employed. The method’s precision was 9%, calculated by analytical blanks coefficient of variation. The recovery of the certificated material was 110%, and the analytical detection limit (DL) was 0.01 ng.

To determine Se content, samples were analysed using inductively coupled plasma optical emission spectrometry (ICP-OES, ICAP 6500 Duo, Thermo Scientific, with One Fast System, ThermoFisher Scientific, Waltham, USA). Previously, the samples were digested in special Teflon reaction tubes with 4 mL of trace mineral-grade nitric acid (69%) and 1 mL of hydrogen peroxide (33%) (Suprapure, Merck) and heated for 20 min at 220 °C in a microwave digestion system (UltraClave-Microwave Milestone®, Sorisole, Italy). Finally, the samples were diluted to 10 mL with double deionised water. Two readings were taken for every sample, so the concentration values used were the mean of the two readings. In order to check any possible contamination, one blank sample for every eleven samples was also analysed. Element calibration standards (SCP Science, in 4% nitric acid) with specific concentrations of this element and intermediate patterns were prepared. The calibration device was established per batch, with a minimum of three points for every lot. Each run started with the calibration standards, continued with samples and intermediate patterns, and finished with the series with intermediate patterns (10% variation coefficient). The wavelengths were 196.090-203.985, and the recovery rates for reference materials (Standard Reference Material L577b) was 103.93%. The DL was 0.001 μg g^−1^.

For a better interpretation of results and to compare between different tissues, the element concentrations of bone were transformed to wet weight (the moisture percentage of each sample were measured, and then the individual THg and Se concentration in wet weight was calculated). Thus, inorganic element concentrations were expressed in micrograms per gram in wet weight (μg g^−1^ ww). 

### 2.3. Data Analysis

The data given for the THg and Se concentrations are mean, standard error, minimum and maximum. The Kolmogorov–Smirnov and Shapiro–Wilk test were used to check the normality of data, and Student’s t-test, ANOVA, Games-Howell (post hoc) and Pearson tests were used as statistical methods. The significance level for all tests was set as 0.05. All statistical analyses were performed with SPSS v.24.0 for Windows.

In terms of food safety, the HBV_Se_ [39] in muscle tissue was calculated:

HBV_Se_ = ([Se – Hg] / Se) × (Se + Hg) (1)

where Se and Hg are molar concentrations (µmol kg^−1^) of these elements. A positive value of HBV_Se_ is considered healthy [39,42]. The scale of the value proportionately reflects the Se deficit or surplus associated with eating that seafood [39]. For better knowledge of Se and Hg relation, we applied this formula to the remaining tissues. The Se/THg concentration ratio and the HBV_Se_ were calculated for each individual sample, then averaged. The Se:Hg molar ratio is not presented (this can be obtained by multiplying the Se/THg concentration ratio by 2.54).

## 3. Results 

The birth-year of bluefin tuna analysed ranged from 2001 to 2006. 

**Figure 1:**
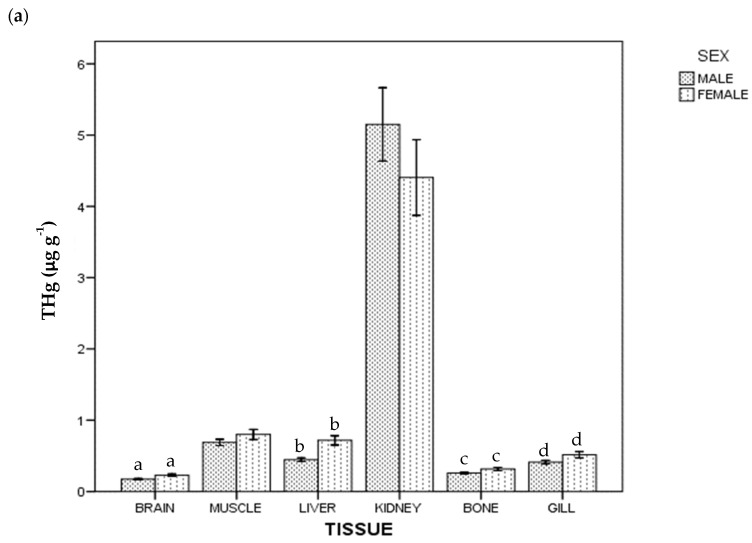

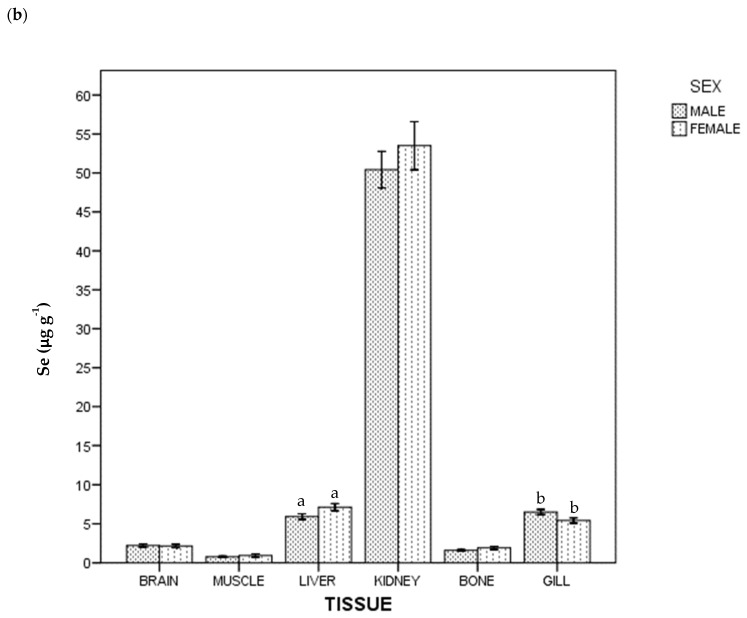
Total Hg (**a**) and Se (**b**) concentrations of ABFT by tissue and sex. For each tissue, the same lowercase letter shows statistical differences between males and females. Bars show standard error. For each tissue, "the same lowercase letter (a, b, c and d in Figure 1a; a and b in Figure 1b) shows statistical differences between males and females”. For example, for liver, “a” is the same lowercase in male and female, so there is statistical differences between them.

Regarding THg and Se concentration, all the samples were above the instrumental DL. The detected concentrations of THg and Se in tissues in whole population are given in Table 1. In general terms, the highest average concentration was of Se in kidney (51.776 µg g^−1^), and the lowest was of THg in brain (0.198 µg g^−1^). Figure 1 shows THg and Se concentrations by tissue and sex: females had a higher THg concentration than males (*p* < 0.05) in brain, liver, bone and gill; for Se, females had a higher concentration than males in liver, but lower in gill (*p* < 0.05).

**Figure 2:**
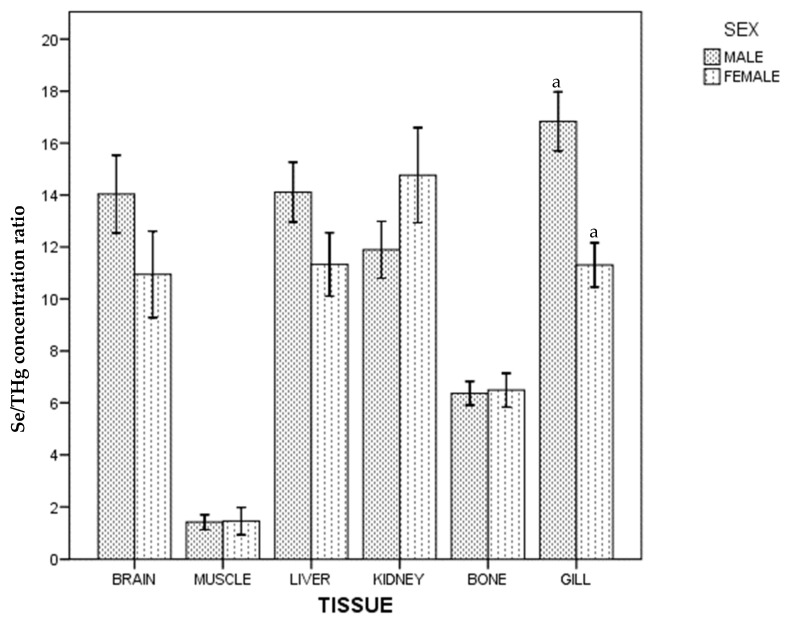
Se/THg concentration ratio of ABFT by tissue and sex. For each tissue, the same lowercase letter shows statistical differences between males and females. Bars show standard error. For each tissue, "the same lowercase letter (a in Figure 2) shows statistical differences between males and females”. For gill, “a” is the same lowercase in male and female, so there is statistical differences between them.

Regarding the whole population, no differences in Se/THg concentration ratio between gill, kidney, liver and brain were found, and these were higher than those found in bone and muscle ((gill~kidney~liver~brain) > bone > muscle). In males, differences between kidney and gill were also found, and no differences between brain and bone in female were found. Differences between sexes are shown in Figure 2.

**Figure 3:**
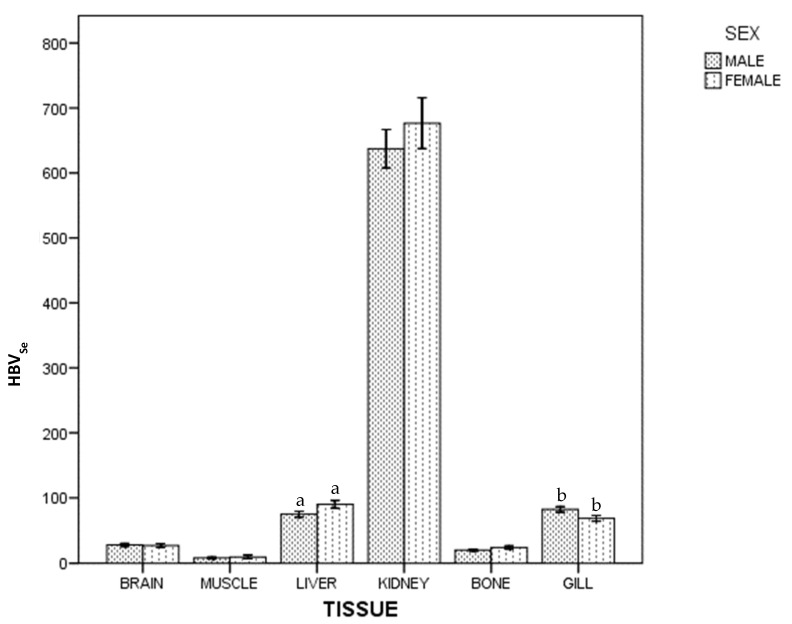
Selenium Health Benefit Value of ABFT by tissue and sex. For each tissue, the same lowercase letter shows statistical differences between males and females. Bars show standard error. For each tissue, "the same lowercase letter (a and b in Figure 3) shows statistical differences between males and females”. For liver, “a” is the same lowercase in male and female, so there is statistical differences between them.

For HBV_Se_, in whole population and by sex, there were statistical differences between tissues: kidney > (liver~gill) > (brain~bone) > muscle. Figure 3 shows differences between females and males: females had a higher HBV_Se_ than males in liver, but lower in gill (*p* < 0.05).

**Figure 4:**
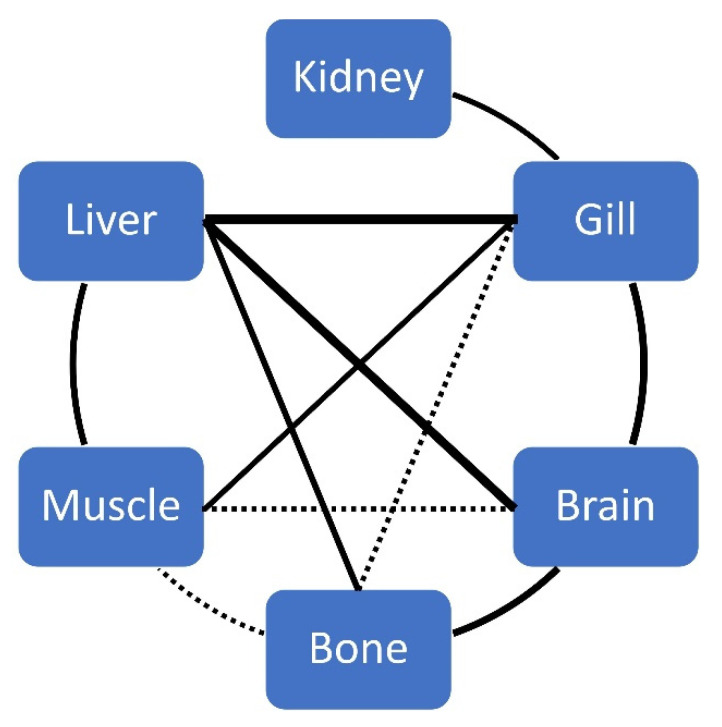
Correlations between tissues for THg concentration in ABFT. The solid line indicates a p-value < 0.01, and a dashed line indicates a p-value < 0.05. The thickness of the line represents the strength of the relationship.

Significant correlations between biometric data, age and elements (by tissue) are given in Table 2. For each element, significant correlations between tissues are given in Table 3 and Table 4, and Figure 4 for Hg.

## 4. Discussion

Mercury is an important element related with the ABFT, and the scientific literature report concentrations of this heavy metal mainly in muscle (Table 5), by food safety. In fact, several studies report the importance of limiting the consumption of this fish, the need of continuous monitoring of Hg levels in this species and the control of tuna size by Hg accumulation [43,44,45]. However, the kinetic of this element in *T. Thynnus* specimens from the Mediterranean Sea is scarce (only Hg concentration in liver was reported). In addition, there are few studies regarding Se and Hg relation in tuna [39,42,46,47,48], and to our knowledge, none in *T. thynnus*. On the other hand, the differences between sex have been scarcely studied [29,48,49]. Thus, better knowledge regarding the distribution, tissue accumulation of THg, and Se concentrations in this species is necessary.

### 4.1. Kidney

The kidney was the tissue with the highest concentration of Se and THg (Table 1), with a high variability for both elements between specimens. This is a well-perfused tissue, so the renal Hg concentration in fish is often high [53,54]. However, kidney is not usually analysed in tuna species, and only one work has reported this data in *T. thynnus* in the Arabian Sea [55], being much lower than those found in our study (0.187 vs. 4.821 µg g^−1^) and in agreement with fish from unpolluted marine water [56]. 

In other tuna species, renal Hg concentrations were also lower than those found in *T. thynnus*: 0.216 µg g^−1^ (*Katsuwonus pelamis*), 0.260 µg g^−1^ (*Thunnus albacares*) and 0.201 µg g^−1^ (*Thunnus obesus*) [57]; even in *T. albacares* and *K. pelamis* the kidney had a mean concentration of 0.39 to 0.45 and 0.20 µg g^−1^, respectively [58]. In some works, the renal Hg concentration has been reported greater than muscle, liver and brain, in the same order of magnitude (e.g., Burger et al. [59], regarding *Pomatomus saltratix*), but kidney of *T. thynnus* (present study) had up to 25 times more Hg than the concentration reported by the cited authors. In this sense, some factors could explain these results: feeding, binding of Hg–protein and sea pollution. First, farm tuna is fed intensively with fish such as sardine (*Sardina pilchardus*), round sardinella (*Sardinella aurita*), scad (*Trachurus trachurus*), Atlantic herring (*Clupea arengus*) or mackerel (Scomber spp.), inter alia, and it is known that MeHg is bioaccumulated in aquatic organisms. According to Raihan et al. [60], the kidney had the highest MeHg concentration, followed by liver and gill, in *Paralichthys olivaceus* fed with a dietary organic Hg; the liver was the tissue in specimens fed with dietary inorganic Hg. On the other hand, an increase in MeHg exposure could accelerate the demethylation in liver [61], binding and immobilizing to metallothionine and other proteins containing sulfhydryl groups [62,63]. The liver’s ability to metabolise or eliminate Hg may determine concentrations in other tissues of the fish [64], and Hg may clear via kidneys as inorganic Hg [65,66]. Metallothionein in fish kidney induced by Hg has been discussed [67], and several studies showed that Hg shifts from low weight thiol proteins to the higher affinity Se containing selenoproteins when there is an increase of dose and time [68], so the target of Hg is Se, not sulphur. 

In our study, there was a high Se concentration in kidney (51.776 ± 1.884 µg g^−1^). In other predator species (*Merluccius productus* and *M. merluccius*), some authors have reported the kidney as the tissue with the highest Se levels, although the concentrations of this element were 11-16 times lower than those found in ABFT [69,70]. Furthermore, a moderate to high relationship between both elements in this tissue (r = 0.531, *p* < 0.01) was found, while this relation was lower in liver (r = 0.395, *p* < 0.01), and a moderate to low relationship between renal THg and Se concentration and fork length were found (Table 2). In addition, the kidney had the highest Se-Hg molar relation (in form of HBV_Se_), followed by the liver (Table 1). Thus, a possible speculation of this high renal concentration of Hg may result from intensive diets of farmed tuna, insufficient ability of liver to store high concentrations of this metal, and an effective Se-based renal protection.

### 4.2. Gills

The concentration of Hg in the tuna gills could be related to the presence of this metal in the Mediterranean Sea, since the studied fish population was caught in this area, and it was kept and fattened in these waters for 4-5 months. Regarding Storelli et al. [32], abiotic factors may affect the metal accumulation in fish, and it is generally believed that the Mediterranean Sea contains high Hg concentrations scattered throughout its basin, with high Hg levels in pelagic and benthic organisms [34,52]. In fact, some authors reported high Hg concentration in gills after water exposure [71], but other authors [72] reported low Hg concentrations in this tissue, indicating that Hg uptake in fish organs is not mainly from water. 

In our study, ABFT had a similar THg concentration in gill than liver, and a significant correlation between gill and kidney was found (Table 3, Figure 4), which could add the transport of Hg through the body to our hypothesis, at least in this area (kidney was correlated only with gill). Furthermore, THg concentration in gill of *T. thynnus* was similar than those reported by Koffi et al. [57] in *T. albacares*, *T. obesus* and *K. pelamis* (0.440, 0.475 and 0.474 µg g^−1^, respectively) from the Atlantic Ocean, but in the present study gill was the tissue with more Hg concentration; there were also significative relationships between gill and remaining tissues (Table 3, Figure 4). 

Finally, Se concentration in gill was high (seven times greater than those reported in the predator species *M. productus* [69]), with a similar Se/THg concentration ratio to that we found in kidney (14:1 vs. 13:1), but lower HBV_Se_ (Table 1), so we could consider this tissue as a temporal store of Hg.

### 4.3. Muscle

The muscle is a tissue usually analysed in Mediterranean ABFT (Table 5), and it is considered as one of the main tissues of Hg accumulation. In this sense, some authors report that this tissue has a negligible elimination of Hg and an efficient uptake from other tissues [72]. In fact, there was a positive correlation between muscle and the rest of tissues, except kidney (Table 3, Figure 4). In *T. thynnus*, muscle had similar Hg concentration to those reported in farmed tuna in several Mediterranean areas, lower than those detected in wild tuna (Table 5), and no significant correlation with biometric measures (Table 2). Several authors have reported positive correlation between Hg muscle concentration and weight and/or length [43,44,48,50,52], but curiously, in these studies fish were wild and farmed tuna with low weight (except than those reported by Milatou et al. [50]). No correlation was reported in tuna of greater weight (Annibaldi et al. [48] in farmed tuna), and a negative relationship was reported by Licata et al. [29] in wild tuna. Some authors have reported an important relation of weight/Hg muscular concentration in tuna above 40 kg [43,44]. According to our results, we agree with Nakao et al. [73], who indicated that THg concentration in full-cycle reared bluefin tuna (*T. orientalis*) does not correspond to its body weight, being quite different to wild tuna, and associated to the rapidly growing fish (tissue mass increase faster than the pollutant input [74]). 

In our study, five specimens had a THg concentration above 1.0 µg g^−1^ (1.032, 1.055, 1.083, 1.277 and 1.603 µg g^−1^). In this sense, Se concentration in ocean fish can alleviate Hg-exposure risk [35,75], so it is considered an element of great importance in terms of food safety. Muscular Se concentration was lower than that reported in farm tuna from the Mediterranean Sea with similar weight [48], and higher than that reported by Di Bella et al. [49] in wild *T. thynnus* (Table 5). In addition, those results were similar or lower than those reported in other tuna species [46,47,76,77,78,79]. In fact, the muscle was the tissue with the lowest concentration of Se, so the distribution observed in our study agreed with that previously reported by Acosta-Lizárraga et al. [69] in other predator species. However, an important concept to consider is the relation of Se/Hg, by the ability of Se to decrease or eliminate the toxic effects of Hg [68,80,81,82], which is relevant in fish muscle. Thus, some works have reported a good proportion between both elements in tuna [39,46,83]. In this sense, the muscle was the tissue with the lowest Se/THg concentration ratio (1.4/1), but the HBV_Se_ was, in each specimen, positive (8.777 ± 1.500, 0.351-44.459), similar to that reported in the same and other tuna species [39,42,46,47,48], or even greater than those reported in small pelagic species [84], and thus safe to consumers.

### 4.4. Liver 

Mercury concentration in liver presented intermediate values among the ones reported in previous studies from the Mediterranean Sea (Table 5). Attending the weight of tuna analysed, our results should be similar to the ones reported by Licata et al. [29] and Srebocan et al. [34], but it is necessary to take into account that there was a negative correlation between tuna weight and THg concentration in the liver (r = -0.326, *p* < 0.05), so we could assume that the hepatic mass also increases faster than the Hg input. Nevertheless, hepatic THg concentrations were higher than those reported in *T. thynnus* by Jaffar and Ashraf [55] in the Arabian Sea and in other tuna species (*T. albacares*, *T. obesus* and *K. pelamis*) in other marine areas (Atlantic, Pacific and Indian Ocean) [47,57,58,85]. Total mercury concentration in liver and gill was strongly correlated (r = 0.719, *p* < 0.01), and non-statistical differences between both tissues were found, so the Hg levels in the Mediterranean basin and a direct transport between gills–liver could explain these data. In addition, Se concentration in liver was also negatively correlated with the tuna weight (r = -0.399, *p* < 0.01); a positive relationship (*p* < 0.01) between Se and THg in liver, and no statistical differences in Se concentration between liver and gill were found. Thus, it seems that there was a similar performance pattern between both elements and tissues. In this sense, Tallandini et al. [86] reported similar kinetic characteristics between both tissues for Se. However, no relationship in Se concentration between liver and gill was found, so a transport of Se to the liver from other fish tissues could have happened. On the other hand, liver Se concentrations were lower than those reported in *T. albacares* and *K. pelamis* [47,58], agreeing in order with those reported by other authors in other predator species (second tissue with the highest concentration [69]). 

The European Union, the world’s second largest trader of fishery and aquaculture products after China [87], promotes the use of fishery and aquaculture side streams. In the present study, the THg accumulation in liver was higher than 0.1 µg g^−1^ in all specimens, so theoretically, and according to the Commission Regulation (EU) 2019/1869 [88], these data should be taken in consideration if liver is used as a by-product source. Nevertheless, and as was the case with other tissues, the liver Se/THg concentration ratio was higher than 10, and the HBV_Se_ was also high (Table 1), with a minimum of 17.543, so these data should be taken into account in regulations regarding substances in animal feed.

### 4.5. Brain

Brain had the lowest THg concentration in ABFT (Table 1). Mercury is neurotoxic, its toxicity is widely reported [89], and the risk in humans has been studied [24]. Thus, many studies in human populations are published, including the relationship of Hg with cardiovascular disease, effects in gastrointestinal tract, kidneys, liver, skin, genotoxicity and cancer [90], autism spectrum disorders, and Alzheimer’s Disease [91,92]; however, information regarding its threat to fish nervous system and their mechanisms is still scarce [93]. In fact, there are few studies regarding Hg concentration in the tuna brain, and to our knowledge, none in *T. thynnus*. Regarding several authors, Hg crosses the blood–brain barrier, reaches the fish brain and produces toxicity [94,95,96]. In other tuna species (*T. obesus*, *T. albacares* and *K. pelamis*), Koffi et al. [57] reported very similar Hg concentrations to those we have found in ABFT (0.162 to 0.186 vs. 0.198 µg g^−1^), while the Hg concentration found in other species varies between 0.02 (*Trichiurus lepturus* [97]) and 1.33 µg g^−1^ (*Carcharhinus limbatus* [98]). Regarding Burger et al. [59], the Se/Hg relation maybe important in tissue such as kidney, liver and brain. In *T. thynnus*, no statistical differences between these tissues (plus gill) in Se/THg concentration were found, and these ratio data were higher (*p* < 0.05) than those found in bone and muscle (Table 1), so we agree with these authors about the possible protection role of Se against Hg levels in important tissues such as the brain. 

On the other hand, a positive and significant correlation in THg concentration between brain and the rest of tissue (except kidney) was found (Table 3, Figure 4). Regarding some authors, the MeHg can be demethylated in the brain, and liver and gill also participate in this process, while muscles store MeHg [99,100,101], which could explain these high correlations (Table 3).

### 4.6. Bone

Bone is reported as the main tissue of the bioaccumulation of metals such as Cu, Zn or Fe [102], mainly by the hydroxyapatite. To the best of our knowledge, no data about Hg concentration in *T. thynnus* bone have been reported to date, so the results were compared first with dorsal spine in the same species [103]; the THg concentration in bone was higher (0.283 vs. 0.11 µg g^−1^). In other fish species, Hg concentrations were very low (mean lower than 0.012 µg g^−1^ [102]), or similar (mean of 0.18 to 0.33 µg g^−1^ [104]) to those found in ABFT. Mercury and Se were not correlated in bone, but THg concentration in this tissue was correlated with remaining tissues, except kidney, being high with brain (r = 0.509, *p* < 0.01). Therefore, a priori, one could think that this is a useful tissue for bioaccumulate, but the low THg concentration seems to indicate that this is a temporal place for accumulation prior to elimination by gills or kidney (the gills have been described as the most important route in Hg elimination in marine fish [105], and the kidney was the tissue with highest THg concentration). Regarding Se concentration, no differences between bone and brain were found (neither in HBV_Se_), but the Se/THg concentration ratio was in the middle of those found in gill, kidney, liver and brain, so further studies could explain the relation between both tissues.

### 4.7. Mercury and Selenium by Sex

In ABFT, differences between sexes have been reported in few studies. Thus, some authors have found no differences in Hg and Se muscle and liver concentrations between females and males [29,48], even if other authors found differences in Se concentration [49]. In this sense, it is interesting to note that a recent review with several species showed that males had higher Hg concentration in muscle than females in eight studies, while the opposite situation was reported in seven studies, concluding that sex is not the main driver of Hg bioaccumulation [106]. On the other hand, in *T. thynnus*, a specific pattern in Se and THg (concentration and distribution) between females and males was not found, even if THg concentration in liver was correlated with brain and gill for both groups (Table 3), and Se and THg concentrations were only correlated in kidney, both in males and females (Table 2). In addition, statistical differences in THg concentration (between sexes) in liver, brain and gill, plus bone, were found (Figure 1a), and only in liver and gill for Se concentration (Figure 1b). This is in agreement with that reported by Madenjian et al. [107], regarding that in teleost fishes, males eliminate Hg faster than females. Finally, regarding tissues, it seems that muscle is the tissue that contributes the least to establishing differences between sexes. 

## 5. Conclusions

The results reported in the present study show an important role of Se against Hg in all tissues analysed, similar in brain, liver, kidney and gill. Total mercury concentration in muscle, remaining soft tissues and hard tissues of ABFT contributes to increasing the knowledge regarding pollution, fish health and food risks for human, but the Se/THg concentration ratio and the HBV_Se_ should be taken into account in each tissue, since high Hg concentrations are important in both meat and by-products of the tuna aquaculture industry. A specific pattern in Se and THg (concentration and distribution) between females and males was not found, and it seems that muscle is the tissue that contributes the least to establishing differences between sexes.

## Figures and Tables

**Table 1:** Concentration of THg and Se (µg g^−1^, ww), Se/THg concentration ratio and HBV_Se_ in tissues of ABFT. Data: mean±standard error, minimum-maximum.

*n* = 43	THg	Se	Se/THg	HBV_Se_
Brain	0.198 ± 0.010 (0.084–0.350)	2.188 ± 0.145 (0.802–4.609)	12.672 ± 1.124 (2.759–32.018)	27.664 ± 1.836 (9.975–58.349)
Muscle	0.737 ± 0.040 (0.089–1.603)	0.847 ± 0.110 (0.293–3.516)	1.435 ± 0.274 (0.414–10.471)	8.777 ± 1.500 (0.351–44.459)
Liver	0.566 ± 0.038 (0.166–1.542)	6.452 ± 0.298 (1.390–12.801)	12.881 ± 0.856 (5.382–28.803)	81.601 ± 3.770 (17.543–162.049)
Kidney	4.821 ± 0.371 (1.636–11.234)	51.776 ± 1.884 (32.910–81.000)	13.162 ± 1.025 (5.362–39.447)	654.663 ± 23.811 (416.305–1022.739)
Bone	0.283 ± 0.013 (0.145–0.531)	1.736 ± 0.106 (0.430–4.565)	6.422 ± 0.379 (1.255–11.668)	21.875 ± 1.349 (4.908–57.731)
Gill	0.458 ± 0.025 (0.187–1.030)	6.024 ± 0.250 (3.194–10.567)	14.391 ± 0.843 (5.329–30.163)	76.216 ± 3.170 (40.345–133.805)

**Table 2:** Statistically significant correlations between biometric data, age and elements concentration in tissues of ABFT; L = liver, K = kidney, Bo = bone, M = muscle, G = gills, Br = brain.

	Fork Length			Weight			Age			THg			Se		
	Whole	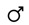	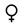	Whole	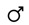	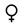	Whole	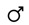	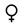	Whole	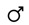	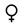	Whole	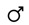	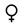
Fork length															
Weight	0.827 **	0.692 **	0.850 **												
Age	0.369 *	0.521 **													
THg	0.330 * K	0.448 * K		−0.326 * L											
Se	0.319 * K	0.477 * Bo	0.484 * K	-0.399 ** L		0.476 * K	−0.305 * M			0.395 ** L 0.531 ** K	0.594 ** K	0.531 * K 0.537 * G			

** Significant correlation at 0.01 level. * Significant correlation at 0.05 level.

**Table 3:** Statistically significant correlations for THg concentration between tissues of ABFT.

	Brain			Muscle			Liver			Kidney			Bone			Gills		
	Whole	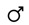	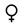	Whole	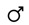	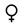	Whole	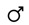	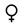	Whole	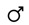	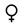	Whole	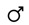	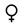	Whole	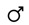	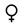
Brain																		
Muscle	0.355 *		0.459 *															
Liver	0.632 **	0.465 *	0.571 *	0.463 **		0.625 **												
Kidney																		
Bone	0.509 **	0.518 **		0.362 *			0.433 **											
Gills	0.532 **	0.531 **		0.403 **		0.550 *	0.719 **	0.420 *	0.816 **	0.403 **		0.665 **	0.371 *	0.519 **				

** Significant correlation at 0.01 level. * Significant correlation at 0.05 level.

**Table 4:** Statistically significant correlations for Se concentration between tissues of ABFT.

	Brain			Muscle			Liver			Kidney			Bone			Gills		
	Whole	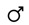	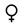	Whole	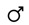	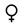	Whole	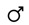	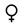	Whole	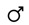	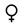	Whole	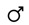	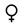	Whole	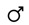	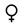
Brain																		
Muscle																		
Liver																		
Kidney					0.522 **													
Bone				0.447 **		0.564 *					0.556 **							
Gills																		

** Significant correlation at 0.01 level. * Significant correlation at 0.05 level.

**Table 5:** Total mercury and Se concentrations in ABFT from Mediterranean Sea. Data: mean ± standard deviation, µg g^−1^, ww.

Location	n	Origin	Weight (Kg)	Tissue	Hg	Se	Reference
South-East Malta	40	Farm	238 ± 93	Muscle	0.61 ± 0.20	1.07 ± 0.86	[48]
Sardinia (Italy)	33	Wild	45 ± 26	Muscle	1.68 ± 0.58	0.64 ± 0.31	
Ionian Sea (Greece)	20	Farm	80–540 (254.9 ± 96.7)	Muscle	0.57 ± 0.21 (0.28–1.28)	na	[50]
Adriatic Sea	29	Farm	100–300	Muscle	0.899 (0.490–1.809)	na	[34]
	15			Liver	1.165 (0.324–3.248)	na	
Strait of Messina (Italy)	14	Wild	50-190	Muscle	3.03 ± 0.55 (2.45–4.21)	na	[29]
				Liver	1.88 ± 0.54 (1.32–3.02)	na	
Ionian Sea	73 (pools, n = 7)	Wild	2.85–4.36 (3.613 ± 0.471)	Muscle	0.20 ± 0.07 (0.13–0.35)	na	[32]
				Liver	0.39 ± 0.10 (0.27–0.60)	na	
Mediterranean Sea (Spain)	3	Wild	0.74–1.085	Muscle	0.207 ± 0.087 (0.144–0.306)	na	[33]
				Liver	0.276 ± 0.005 (0.270–0.280)	na	
Mediterranean Sea (Italy)	23	Wild	130–290	Muscle	0.446 (0.246–0.714)	0.607 (0.270–1.207)	[49]
Tyrrenian Sea	169	Wild	39.5 ± 43.8 (0.33–158)	Muscle	1.02 ± 0.99 (0.07–4.26)	na	[43]
Ionian Sea	161 (14 pools)	Wild	36.1 ± 23.53 (5.3–83)	Muscle	1.18 ± 0.85 (0.16–2.59)	na	[44]
Aegean Sea (Turkey)	6	Farm	91.5 ± 2.59	Muscle	0.454 ± 0.048	na	[45]
	43		235.60 ± 75.53	Muscle	0.490 ± 0.064	na	
Tyrrhenian Sea	20	Farm	56.4 ± 34.0 (13–161)	Muscle	0.61 (0.07–1.76)	na	[51]
Mediterranean Sea (Italy)	23	Farm	135.7 ± 45.8 (46.5–258.8)	Muscle	0.660 ± 0.585 (0.140–2.211)	na	[52]

## Data Availability

Data sharing not applicable.
